# Pain in the body, harm to the heart: advances in research on the impact of chronic pain on cardiovascular diseases

**DOI:** 10.3389/fcvm.2025.1629145

**Published:** 2025-08-06

**Authors:** Yanli Zhang, Xiaochen Wu, Feng Gao, Jian Wang

**Affiliations:** Department of Cardiovascular Surgery, The General Hospital of Western Theater Command, College of Medicine, Southwest Jiaotong University, Chengdu, Sichuan, China

**Keywords:** chronic pain, cardiovascular diseases, pathogenesis, treatment, comorbidity

## Abstract

Chronic pain (CP) is highly prevalent, and a substantial proportion of patients concurrently suffer from cardiovascular diseases (CVD), suggesting a complex interplay between these two conditions. CP increases the risk of CVD through multiple mechanisms, including sympathetic overactivation, neuroimmune inflammatory responses, endocrine and metabolic dysregulation, and bidirectional regulation along the heart-brain axis. Moreover, pharmacological treatments traditionally used for CP, such as non-steroidal anti-inflammatory drugs (NSAIDs), opioids and related agents, are associated with heightened cardiovascular risks. In contrast, non-pharmacological interventions like spinal cord stimulation and cognitive behavioral therapy have demonstrated dual potential in alleviating pain and improving cardiovascular outcomes. There is an urgent need to focus on the comorbid mechanisms linking CP and CVD, to develop precise and individualized intervention strategies, and to integrate pain management into cardiovascular prevention and treatment frameworks, thereby optimizing interdisciplinary approaches to comorbidity prevention and care.

## 1 Introduction

Chronic pain (CP), as defined by the International Association for the Study of Pain (IASP), refers to pain that persists or recurs for more than three months, and its development involves a complex interplay of biological, psychological, and social factors (1). As a chronic condition, CP is increasingly recognized to be closely associated with various cardiovascular diseases (CVD). Emerging evidence suggests that CP serves as a significant risk factor for the onset and progression of multiple types of CVD (2–6). A large-scale study involving 475,000 adults in the United Kingdom revealed that approximately 8.6% of individuals with CP concurrently suffered from CVD, highlighting a strong association between CP and adverse cardiovascular events (2). Several reports have indicated that patients with chronic chest pain and those with CVD exhibit more frequent ST-segment depression on electrocardiograms and an earlier decline in exercise tolerance, suggesting potential shared pathophysiological mechanisms (7, 8). Functional imaging studies further demonstrated that patients with coronary artery disease and angina exhibit abnormal activation patterns in brain regions such as the anterior cingulate cortex and the insular cortex, which closely resemble those observed in CP patients (9). These findings suggest that cardiac pain may also involve central sensitization mechanisms. Collectively, current research indicates that CP may influence CVD development through multiple pathways.

CP not only contributes to the initiation of CVD but may also deteriorate its long-term prognosis. Globally, CVD is responsible for approximately 17.9 million deaths annually, accounting for 31% of all deaths (10), with 8%–11.2% of these patients concurrently suffering from CP, thereby imposing a substantial medical and socioeconomic burden (11). Notably, CP is characterized by recurrent episodes, chronic persistence, and refractory, often resulting in significant patient suffering. The intractability of CP is due to not only the complex underlying mechanisms itself but also from the presence of numerous pain-related comorbidities, such as anxiety, depression, CVD, and various forms of functional impairments or disabilities. CP and CVD frequently interact and exacerbate each other, forming a vicious cycle that renders pain symptoms more refractory and lack of responsive to conventional treatments. Therefore, pain management should not solely focus on the perceptual aspects of CP, such as simply reducing pain intensity or frequency. More importantly, a holistic approach is required that comprehensively assesses and simultaneously addresses co-occurring conditions, particularly the adverse impacts on the cardiovascular system. These comorbidities not only aggravate the overall disease burden but also significantly increase the complexity and cost of treatment. In this context, there is an urgent need to shift pain management strategies from a symptom-centered, single-discipline model to an integrated, multidisciplinary intervention framework. Only by achieving improvements in both the core manifestations of CP and its associated CVD can the quality of life for CP patients be meaningfully enhanced. This review aims to elucidate the mechanisms by which CP influences the development and progression of CVD. Moreover, the potential of pain management strategies to improve cardiovascular outcomes are reviewed, thereby providing new theoretical foundations and intervention targets for cardiovascular risk management.

## 2 Method

An integrative review synthesizes existing studies to identify connections and gaps in the literature, stimulate new ideas, and guide future research. This review focuses on the impact of CP on CVD. Building on search strategies used in previous studies (blinded for review), the literature has been updated to include recent findings from both basic and clinical research, with the aim of mapping current evidence and proposing new directions for investigation.

### 2.1 Systematic literature search

A systematic literature search was performed in November 2024, covering publications from January 2000 through November 2024. The electronic databases searched included PubMed and Web of Science, and Cochrane Central Register of Controlled Trials. A combination of truncated search terms and Boolean logic was applied. Search terms included: (chronic pain* OR persistent pain* OR neuropathic pain* OR nociplastic pain*) AND (cardiovascular disease* OR CVD* OR myocardial infarction* OR heart failure* OR ischemia* OR hypertension* OR atheroscleros* OR autonomic dysregulation* OR endothelial dysfunction* OR heart rate variability* OR sympathetic* OR inflammation*). Search results were filtered to peer-reviewed journal articles, abstract/title/keywords, full text availability, English language, 2000–2024 publications, relevant subjects, and references available. The search results were stored and managed using Endnote 21 and Zotero.

**Figure 1:**
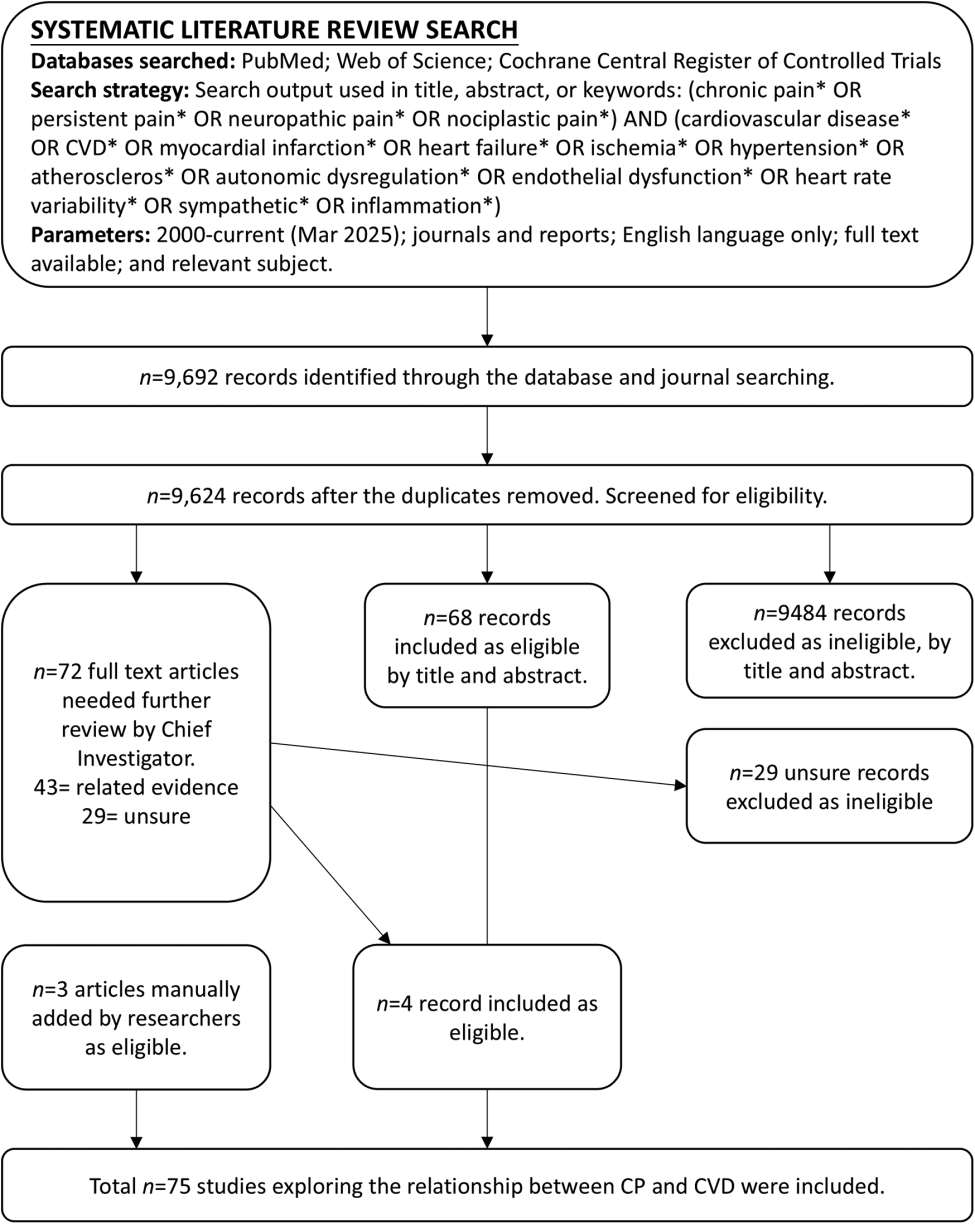
PRISMA flow diagram for the systematic literature review. Excluded = did not explore the relationship between CP and CVD; Included = meets the inclusion criteria; Related evidence = did not directly examine the CP-CVD relationship but includes relevant data of potential significance; Unsure = insufficient information in the title and abstract; full-text article was reviewed for clarification.

Articles were eligible for inclusion if they: (1) provided empirical evidence (quantitative or qualitative) on the association between CP and CVDs or related risk factors; (2) studied adult or adolescent human populations; (3) were full-text accessible in English; and (4) were original research articles published in peer-reviewed journals. After removing duplicates, titles and abstracts were screened, followed by full-text assessment against inclusion criteria. A total of 9,692 records were retrieved. After removing duplicates *(n* = 68), 9,624 records remained for screening based on the inclusion criteria. Records that did not meet the inclusion criteria were excluded. In total, 75 journal articles met the inclusion criteria. Figure 1 presents the PRISMA flow diagram illustrating the multi-step process of inclusion and exclusion.

### 2.2 Targeted literature review

To identify policy reports and surveillance data not indexed in academic databases, targeted grey literature retrieval was performed in November 2024. Sources included: the World Health Organization (WHO), Centers for Disease Control and Prevention (CDC), Global Burden of Disease (GBD) Study database, National Institutes of Health (NIH), IASP, and American Heart Association (AHA). The same search strategy and time frame (2000–2024) were applied. Reports and research briefs were screened for relevance based on title, content, and empirical contribution.

### 2.3 Updating

In March 2025, a time-limited supplemental search was executed to capture emergent evidence. This update identified two eligible empirical studies published between January and February 2025. These studies, identified via targeted alerts and hand-searching of key journals, reflect ongoing research interest in the intersection of CP and CVDs and were incorporated into the final evidence synthesis.

### 2.4 Analytical approach

All included publications were reviewed to extract key findings, concepts, and methodological characteristics. A thematic synthesis approach was used to classify studies into the following categories: (1) epidemiological patterns and comorbidity profiles; (2) biological and physiological mechanisms linking CP and CVDs (e.g., sympathetic activation, inflammation, HRV dysregulation); (3) translational Clinical Therapeutics and Future Directions. Literature beyond the inclusion criteria was selectively referenced to contextualize key mechanisms or theoretical perspectives. This integrative analysis facilitated the identification of research gaps and the formulation of new conceptual linkages between chronic pain and cardiovascular vulnerability.

## 3 Epidemiological characteristics and pathogenesis of CP

Current statistics indicate that approximately 20%–30% of the global population suffers from CP (12). Studies have reported a CP prevalence of 18% in developing countries (13), whereas the prevalence is even higher in developed countries, ranging between 10% and 50% (14). CP is commonly characterized by persistent pain, allodynia, sensory abnormalities, hyperalgesia, and dysesthetic pain. These symptoms often lead to sleep disturbances, emotional changes, and functional impairments, significantly diminishing patients' quality of life.

Contemporary research has identified peripheral sensitization, central sensitization, and neuron-glia interactions as pivotal mechanisms underpinning the chronification of pain. Peripheral sensitization involves an enhanced responsiveness and a lowered activation threshold of peripheral nociceptors. During tissue injury or inflammation, inflammatory mediators such as prostaglandins, bradykinin, and interleukins (ILs) activate signaling pathways like protein kinase A and protein kinase C, leading to the upregulation and functional enhancement of ion channels such as TRPV1 and Nav1.8. This process renders normally innocuous mechanical or thermal stimuli capable of evoking pain, a phenomenon that, while initially protective, may promote CP if sustained (15). Central sensitization refers to the amplification of neural signaling within the central nervous system, resulting in pain hypersensitivity. This phenomenon is associated with excessive activation of N-methyl-D-aspartic acid (NMDA) receptors, impairment of inhibitory neurotransmission [notably involving γ-aminobutyric acid (GABA) and glycine systems], and synaptic plasticity alterations (16). Long-term potentiation like mechanisms at the spinal level have also been implicated in maintaining the heightened sensitivity observed in CP states. In addition to neuronal mechanisms, glial cells, particularly microglia and astrocytes, have emerged as critical contributors to the persistence of CP (17). Activated by neuronal signals such as ATP and fractalkine, glial cells release pro-inflammatory cytokines (e.g., IL-6, TNF-α) and neurotrophic factors [e.g., brain-derived neurotrophic factor (BDNF)], which further potentiate neuronal excitability (18). Furthermore, glial modulation of glutamate metabolism and synaptic remodeling disrupts neuroimmune homeostasis, creating a positive feedback loop that perpetuates pain hypersensitivity. Collectively, the chronification of pain reflects not only the persistence of peripheral nociceptive inputs but also maladaptive neuroplastic changes within the central nervous system and the neuroimmune interface. These complex and interwoven mechanisms contribute to the refractory nature of CP and thus lack of effective therapies.

## 4 Association between CP and CVD risk

The World Health Organization (WHO) has formally defined CP as an independent disease entity rather than merely a symptom secondary to other diseases. Recent studies have demonstrated that CP is not only closely associated with mental health disorders but is also significantly correlated with CVD. However, the quantitative relationships between the duration and distribution of pain and the risk of CVD have long lacked systematic support from large-scale population-based studies. A team utilized prospective data from the UK Biobank, comprising 475,171 adult participants, to construct the first comprehensive model linking pain characteristics with cardiovascular outcomes, including myocardial infarction (MI), heart failure, stroke, and cardiovascular mortality (2). The study revealed that, compared to pain-free individuals, those suffering from chronic localized pain exhibited a significantly elevated risk of CVD, with an even greater risk observed in individuals with chronic widespread pain. Notably, this study was the first to systematically demonstrate a dose-response relationship between the extent and duration of pain and CVD risk. Namely, the broader the pain distribution and the longer its duration, the higher the risk of CVD. These findings provide new epidemiological evidence supporting CP as an independent risk factor for CVD.

Another study further highlighted that chronic widespread pain is not only significantly associated with all-cause mortality but also positively correlated with cardiovascular mortality (19). These findings underscore that CP is not merely a major determinant of reduced quality of life but may also represent an independent risk factor for CVD. Moreover, the study showed that the population-attributable risk of CP for CVD is comparable to that of diabetes, suggesting that CP may be as influential as diabetes in the development of CVD. Nevertheless, most novel CVD prevention and management guidelines have yet to recognize CP as a cardiovascular risk factor, potentially leading to neglect and undertreatment of cardiovascular risk among CP patients. Given the generally healthier status of the UK Biobank cohort, the true impact of CP on CVD risk in the general population may be underestimated. In light of these findings, future strategies for both primary and secondary prevention of CVD should include enhanced cardiovascular monitoring in individuals with CP and the development of personalized interventions, particularly in the area of pain management.

## 5 Potential mechanisms linking CP to CVD

### 5.1 Necroptosis and oxidative stress

Recent animal studies have shown that CP markedly increases the heart's vulnerability to myocardial ischemia/reperfusion injury (20, 21). In neuropathic pain models, animals display larger infarct sizes, decreased left ventricular ejection fraction, and elevated markers of cardiac injury such as lactate dehydrogenase and creatine kinase-MB isoenzyme (20, 21). Mechanistically, CP enhances TNF-α production and activates the receptor-interacting protein kinase 1-receptor-interacting protein kinase 3 (RIP1-RIP3) complex, which in turn phosphorylates mixed lineage kinase domain-like protein (MLKL) and calcium/calmodulin-dependent protein kinase II (CaMKII). This signaling cascade triggers necroptosis in cardiomyocytes, thereby worsening myocardial susceptibility to ischemia/reperfusion damage (20). At the same time, CP elevates levels of 4-hydroxynonenal in the heart, causing protein carbonylation that notably inactivates sirtuin 1 (SIRT1) and disrupts the liver kinase B1-adenosine monophosphate-activated protein kinase (LKB1-AMPK) protective signaling pathway (21).

Treatment with melatonin can inhibit the RIP3-MLKL/CaMKII necroptotic pathway, reduce oxidative stress, and exert both analgesic and cardioprotective effects (20). Activating aldehyde dehydrogenase (ALDH2) via Alda-1 or adeno-associated virus serotype 9-mediated gene delivery decreases 4-hydroxynonenal in the heart accumulation, restores SIRT1 function, reactivates AMPK signaling, and alleviates cardiac injury related to CP (21). Together, these findings highlight the RIP3/MLKL/CaMKII necroptosis axis and the ALDH2/SIRT1/AMPK oxidative stress pathway as important links between CP and increased cardiovascular risk.

### 5.2 Sympathetic overactivation

Patients with CP often have overactive sympathetic nervous system (SNS) responses and poor autonomic control of the heart and blood vessels. This imbalance plays an important role in causing CVD. In CP, constant SNS activation raises blood norepinephrine (NE) levels. This speeds up the heart rate, causes blood vessels to tighten, and leads to long-term high blood pressure. NE causes vasoconstriction by stimulating α-adrenergic receptors on blood vessel walls. This increases resistance in the blood vessels, worsens high blood pressure, and raises the risk of serious heart and organ damage (22). NE also activates β-adrenergic receptors, which increases how strongly the heart contracts and raises its oxygen demand. This puts the heart under constant stress. As a result, myocardial ischemia may occur, coronary artery spasms may worsen, and the risk of acute coronary syndrome (ACS) increases. Long-term sympathetic activation affects not just blood vessel tone and heart function, but also causes oxidative stress. This damages endothelial cells, speeds up atherosclerosis (AS), and further raises the risk of cardiovascular disease.

In parallel, CP patients often have reduced parasympathetic activity, showing a shift toward sympathetic dominance (23). This autonomic dysregulation is manifested by significantly reduced heart rate variability (HRV), a known predictor of adverse cardiovascular outcomes. The combination of sympathetic overactivation and vagal suppression diminishes cardiac adaptability to autonomic stimuli, increasing the risk of cardiovascular events, arrhythmias, and sudden cardiac death. Interestingly, even though overall vagal tone is low, CP can increase local parasympathetic activity and raise M2 receptor expression in atrial muscle cells. This causes changes in heart electrical signals, such as longer PR intervals and shorter refractory periods, making atrial fibrillation more likely (24). Notably, this effect can be fully reversed by atropine, an M2 receptor antagonist, implicating aberrant local parasympathetic activation as a key mechanism in atrial fibrillation pathogenesis (24). Therefore, in CP patients, heart risk is linked both to overall autonomic imbalance and abnormal local vagal activation that disturbs heart rhythm stability.

Chronic overactivity of the SNS can disrupt the renin-angiotensin-aldosterone system (RAAS). When RAAS is overactivated, it causes blood vessels to constrict and the body to retain salt and water, leading to high blood pressure and forming a harmful cycle. In CP patients, RAAS problems put extra strain on the heart and blood vessels, raising the risk of heart enlargement, heart failure, and stroke. Clinical studies show that people with CP have higher systolic and diastolic blood pressure than those without pain (25). This is likely due to long-term SNS overactivity. For example, patients with fibromyalgia—a common type of CP—show strong sympathetic activation and ongoing low-grade inflammation. Compared to healthy people, they have a much higher risk of ischemic heart disease, likely caused by SNS related blood vessel dysfunction, poor microcirculation, and lasting effects of chronic inflammation (26).

### 5.3 Inflammatory response

#### 5.3.1 Neuroinflammation maintains and amplifies CP

CP can cause long-lasting systemic inflammation, which increases the risk of CVD. CP activates both peripheral and central inflammation pathways, leading to the release of pro-inflammatory cytokines. This speeds up AS and damages the blood vessel lining. Studies show that people with CP have higher levels of inflammatory markers like IL-6, TNF-α, and C-reactive protein. These cytokines not only affect pain-sensing neurons directly but also influence how the nervous and immune systems interact, making pain worse and more persistent. Abnormal cytokine expression plays a key role in both neuropathic and inflammatory pain (27, 28). For example, it has been demonstrated that Schwann cells, sensory neurons, and microglia interact via the CNTF-STAT3-IL-6 pathway in neuroinflammation. After a peripheral nerve injury, Schwann cells release ciliary neurotrophic factor, which activates STAT3 in sensory neurons and causes them to release IL-6. The IL-6 then activates microglia in the spinal cord, triggering a chain of neuroinflammatory responses. This shows that CNTF, besides helping neurons grow, may also act as a damage signal that promotes CP (27). Furthermore, it has been reported that IL-6 is a key factor in neuropathic pain. It works through the JAK/STAT, MAPK/ERK, and PI3K/Akt pathways to increase pain sensitivity (28). In many pain models, IL-6 levels are much higher in the spinal cord and dorsal root ganglia. Giving animals IL-6 causes mechanical allodynia and thermal hyperalgesia. In contrast, injecting anti-IL-6 antibodies into the spine greatly reduces these symptoms. This suggests that IL-6 could be a useful treatment target for CP.

#### 5.3.2 Systemic inflammation promotes endothelial dysfunction

These inflammatory mediators involved in CP also contribute significantly to cardiovascular pathology. First, long-term release of cytokines such as TNF-α and IL-6 can impair endothelial cell function by reducing nitric oxide (NO) production, leading to decreased vasodilation, vascular stiffness, and impaired blood flow regulation. Damage to the structure and function of endothelial cells is an early and important step in AS. It helps fats and immune cells build up in the vessel wall (29). Second, Inflammation also promotes the oxidation of low-density lipoprotein, which accelerates plaque formation and increases the risk of vascular occlusion. IL-6 and C-reactive protein are key players in this process. High levels of these markers are linked to unstable plaques in the coronary arteries, showing that systemic inflammation contributes to sudden heart events (30). Unstable plaques are more likely to rupture, which can cause heart attacks or strokes (31). Third, chronic inflammation enhances platelet reactivity and coagulation, raising the risk of thrombosis. CP related inflammation may upregulate clotting factors and promote platelet aggregation, especially in individuals with pre-existing AS. C-reactive protein can directly help platelets stick together, and IL-6 increases clotting by controlling how the liver makes certain proteins during inflammation (32). Comorbid inflammatory diseases such as gout and rheumatoid arthritis (RA) further elevate cardiovascular risk through sustained inflammation and a prothrombotic state, as observed in the higher incidence of heart disease and venous thromboembolism in these populations (33). These findings underscore the need to integrate cardiovascular risk management into the care of patients with chronic inflammatory conditions (34). In conclusion, inflammation may be the key link between CP and CVD. It contributes to problems like endothelial damage, plaque formation, plaque rupture, and blood clot formation.

### 5.4 Endocrine and metabolic dysregulation

CP is not just a local injury or abnormal sensory response. It is a systemic disease involving problems in the neuroendocrine-immune system. One key mechanism is the long-term activation of the hypothalamic-pituitary-adrenal axis. Ongoing pain acts like a stress signal, triggering the hypothalamic-pituitary-adrenal axis to release ACTH. This leads the adrenal glands to produce glucocorticoids, especially cortisol. Short-term increases in cortisol help control immunity and keep blood pressure and sugar levels stable. But when cortisol stays high for too long, it causes serious metabolic problems and harms the heart and blood vessels. On one hand, high cortisol levels are linked to insulin resistance (IR), which is a major cause of type 2 diabetes, metabolic syndrome, and AS (35). Long-term high cortisol levels reduce insulin sensitivity in several ways. They block insulin signaling, increase fat breakdown, and raise levels of free fatty acids (FFAs) in the blood. FFAs then make it harder for muscles and the liver to use glucose, creating a cycle of IR, fat toxicity, and metabolic imbalance. On the other hand, IR along with abnormal blood lipids, speeds up coronary artery disease. Patients with CP frequently exhibit lipid abnormalities, including elevated plasma triglycerides, reduced high-density lipoprotein cholesterol, and an increased proportion of small dense low-density lipoprotein particles. Small dense low-density lipoprotein is particularly atherogenic due to its high susceptibility to oxidation and enhanced endothelial permeability. Macrophages absorb them more easily, forming foam cells—an early and important step in plaque formation (36, 37). Simultaneously, FFAs promote hepatic synthesis of very low-density lipoproteins and activate cholesteryl ester transfer protein, resulting in lipoprotein remodeling and a pro-atherogenic lipid profile. More importantly, IR further contributes to CVD risk via multiple mechanisms, including the promotion of hypertension, prothrombotic states, and systemic low-grade inflammation (35). First, IR reduces NO production in blood vessels, which weakens vasodilation. Second, FFAs increase reactive oxygen species, which destroy NO and cause blood vessels to constrict. Third, IR activates the SNS and the RAAS, leading to sodium retention and high blood pressure. IR also increases blood thickness and raises clotting factors, causing a pro-thrombotic state. In addition, fat tissue in IR conditions releases inflammatory cytokines like TNF-α and IL-6. These promote inflammation and damage to blood vessel walls, helping plaque form. In short, CP causes long-term activation of the hypothalamic-pituitary-adrenal axis and overproduction of cortisol. This leads to IR and abnormal blood lipids. Together, these changes disrupt hormone and metabolic systems and raise the risk of CVD.

### 5.5 Other relevant mediators

In the context of CP, the dysregulated release of various neurogenic bioactive substances also poses potential threats to the cardiovascular system. Calcitonin gene-related peptide (CGRP), a key mediator in nociceptive signal transmission, is widely expressed in both central and peripheral sensory nerve terminals and exerts potent vasodilatory and neuromodulatory effects (38, 39). During acute pain states, CGRP contributes to vascular protection by activating endothelium-dependent NO signaling pathways, thereby maintaining microcirculatory homeostasis (40). However, under CP conditions, sustained overexpression of CGRP can lead to disrupted vascular smooth muscle cell responsiveness to vasoactive signals, endothelial dysfunction, and increased vascular permeability, which collectively facilitate monocyte infiltration and chronic vascular inflammation (41). Both animal models and clinical studies have demonstrated significant associations between elevated CGRP levels and pathological vascular remodeling conditions such as AS and hypertension (42–44). Moreover, CGRP can enhance platelet activation and aggregation by upregulating the expression of purinergic P_2_Y_12_ receptors on platelet surfaces, thus playing a critical role in CP related thrombogenesis. In addition to CGRP, other neuropeptides—including substance P and BDNF—serve as important molecular bridges between CP and cardiovascular injury (45). Substance P acts primarily through activation of the neurokinin 1 receptor, inducing both vasodilation and plasma extravasation, while also promoting neutrophil recruitment and stimulating the release of pro-inflammatory cytokines, thereby exacerbating endothelial inflammatory responses (46). BDNF, on the other hand, modulates sympathetic synaptic plasticity, leading to excessive NE release (47). This, in turn, promotes proliferation and migration of vascular smooth muscle cells, contributing to vascular remodeling. Furthermore, BDNF directly acts on cardiac fibroblasts within myocardial tissue, stimulating fibrotic processes that reduce myocardial compliance and impair diastolic function. These pathological alterations are implicated in the development of hypertension and, with chronic progression, may culminate in end-stage cardiovascular events such as heart failure (48).

It is noteworthy that the aforementioned mediators possess dual neuro-immune regulatory properties and, under CP conditions, can engage in a positive feedback loop with heightened sympathetic nervous activity and the local inflammatory microenvironment. This feedback amplification exacerbates oxidative stress and endothelial dysfunction, ultimately contributing to irreversible cardiovascular damage. Moreover, migraine—particularly with aura—has been recognized as an important CVD risk factor among younger populations. Studies suggest that potential mechanisms include cortical spreading depression-induced cerebral vasospasm and CGRP mediated vascular inflammation and thrombogenesis. Additionally, migraineurs frequently exhibit a hypercoagulable state, arterial endothelial dysfunction, and significant blood pressure variability, all of which may further increase the likelihood of CVD occurrence.

### 5.6 The heart-brain axis

The association between CP and CVD may be closely linked to the bidirectional interactions within the heart-brain axis. This axis refers to a complex neuroendocrine-immune regulatory network between the heart and the brain that plays a central role in maintaining physiological homeostasis and modulating pathological states. Recent studies employing viral tracing techniques—such as retrograde trans-synaptic pseudorabies virus and anterograde trans-synaptic herpes simplex virus —have elucidated the pathways and hierarchical regulatory patterns governing heart-brain communication (49). Pseudorabies virus injection into the myocardium retrogradely labels multiple key regions of the central nervous system, including the hypothalamus, cortex, and medulla, revealing a top-down hierarchical control network. This network regulates cardiovascular functions such as heart rate, blood pressure, and myocardial contractility, and is critically involved in stress responses, inflammation, and sympathetic modulation. Conversely, herpes simplex virus injected into the myocardium anterogradely traces afferent cardiac signaling pathways to central areas including the brainstem and cerebral cortex, enabling the brain to perceive and process information regarding cardiac status. This bidirectional communication ensures dynamic adjustment between the brain and heart across physiological and pathological states. Under CP conditions, persistent sympathetic overactivation and sustained release of proinflammatory mediators can disrupt autonomic balance, impairing cardiovascular regulation. Moreover, chronic inflammatory states may drive maladaptive neuroplasticity within central functional networks—such as the prefrontal cortex, limbic system, and insular cortex—affecting pain perception, emotional regulation, and cardiovascular control (50).

## 6 Management of CP comorbid with CVD

### 6.1 Nonsteroidal anti-inflammatory drugs (NSAIDs)

CP significantly increases the risk of CVD through mechanisms such as sustained SNS activation, chronic low-grade inflammation, and alterations in lifestyle behaviors. Therefore, an appropriate pain management strategy not only alleviates pain but may also contribute to cardiovascular protection by reducing sympathetic activity, attenuating inflammation, and improving hemodynamic stability. NSAIDs are commonly used analgesics in clinical practice; however, their impact on the cardiovascular system is complex and bidirectional. For instance, aspirin, a classical NSAID, plays a pivotal role in the secondary prevention of cardiovascular events. Substantial evidence demonstrates that aspirin effectively reduces the incidence of MI and stroke in high-risk populations (51). Its primary mechanism involves the inhibition of cyclooxygenase-1 (COX-1), which reduces thromboxane A₂ synthesis, thereby inhibiting platelet aggregation and decreasing the risk of atherosclerotic plaque rupture and thrombus formation (52, 53). Thus, in CP patients with coexisting CVD risk factors, low-dose aspirin may provide additional cardiovascular protection alongside its analgesic effects. However, not all NSAIDs confer cardiovascular benefit. Long-term use of certain NSAIDs, such as diclofenac and etoricoxib, has been associated with increased risks of hypertension, MI, and stroke (54). This adverse profile may be linked to their selective inhibition of cyclooxygenase-2 (COX-2), which reduces the synthesis of prostacyclin (PGI₂), a vasodilatory and antithrombotic prostanoid. The suppression of PGI₂ may lead to vasoconstriction, elevated blood pressure, and enhanced thrombogenic potential. Consequently, in the long-term management of CP, it is critical to balance the analgesic efficacy of NSAIDs against their potential cardiovascular risks. For patients with high cardiovascular risk, safer alternatives such as acetaminophen or short-term, low-dose NSAID regimens should be prioritized to minimize the risk of adverse cardiovascular outcomes.

### 6.2 β-adrenergic receptor blockers

In the therapeutic strategy for CP comorbid with CVD, β-adrenergic receptor blockers possess a unique dual therapeutic potential owing to their sympatholytic properties. By antagonizing the binding of NE and epinephrine to β-adrenergic receptors, these agents attenuate sympathetic excitatory signaling, thereby modulating cardiovascular dysregulation while potentially addressing autonomic dysfunction associated with CP. Excessive sympathetic activation is commonly observed in CP patients, manifesting as reduced HRV, sustained peripheral vasoconstriction, and elevated circulating NE levels. These factors not only exacerbate pain perception but may also increase the risk of triggering or aggravating cardiovascular events. β-blockers alleviate cardiovascular burden by lowering heart rate, reducing myocardial oxygen consumption, and improving HRV. Moreover, evidence suggests that they may mitigate pain sensitization under sympathetic dominance by reducing peripheral sympathetic outflow and central NE activity. Clinically, non-selective β-blockers such as propranolol have demonstrated analgesic effects in conditions characterized by chronic widespread pain, including fibromyalgia. These effects are hypothesized to involve central modulation of SNS activity. A randomized, double-blind, crossover study evaluated the efficacy of low-dose propranolol in female patients with fibromyalgia and temporomandibular joint disorder, revealing significant reductions in pain scores, thereby supporting the role of β-blockers in attenuating pain via sympathetic modulation (55). Furthermore, an ongoing trial aims to assess the feasibility of low-dose propranolol (10 mg and 20 mg twice daily) compared with placebo in fibromyalgia patients, reflecting growing interest in the utility of β-blockers in CP management. In patients with osteoarthritis, β-blocker use has also been associated with a lower incidence of joint pain and reduced opioid consumption, further supporting their potential benefits in CP contexts (56). In addition, β-blockers may help alleviate CP related comorbidities such as sleep disturbances, anxiety, and depression, thereby indirectly improving both cardiovascular health and pain control. Compared with NSAIDs, β-blockers present a more favorable cardiovascular safety profile, making them particularly suitable for CP patients with a history of hypertension, arrhythmias, or coronary atherosclerotic heart disease. Therefore, in the comprehensive management of CP with coexisting CVD, β-blockers not only serve as a cornerstone of cardiovascular therapy but also offer a viable option for modulating pain and sympathetic overactivation, providing a multi-targeted intervention strategy.

### 6.3 Pharmacological therapies targeting the CGRP receptor

CGRP is a potent vasodilatory neuropeptide that is widely distributed in both the peripheral and central nervous systems, with particularly abundant expression within the trigeminal system. Under physiological conditions, CGRP contributes to cardiovascular protection by activating the CRLR/RAMP1 receptor complex expressed on vascular endothelial and smooth muscle cells, thereby mediating vasodilation, enhancing blood flow regulation, and mitigating oxidative stress (40). However, under certain pathological conditions, excessive CGRP release may induce cerebral vasodilation, neurogenic inflammation, and nociceptive sensitization, establishing CGRP as a key mediator in the pathogenesis of migraine attacks (41). Based on this mechanistic target, a series of monoclonal antibodies directed against CGRP or its receptor have been approved in recent years for the preventive treatment of migraine (57, 58). Multiple clinical trials have demonstrated that these agents significantly reduce the frequency and intensity of migraine episodes. Compared to traditional NSAIDs, CGRP-targeted monoclonal antibodies exhibit greater specificity and a lower risk of cardiovascular side effects (59, 60). Nevertheless, given that CGRP exerts cardioprotective effects—such as promoting vasodilation, reducing oxidative stress, and inhibiting apoptosis—long-term inhibition of its activity may compromise this physiological defense mechanism and potentially elevate the risk of ischemic cardiovascular events. Therefore, despite the demonstrated efficacy and tolerability of CGRP monoclonal antibodies in CP management, concerns remain regarding their safety in patients with coexisting CVD. Current clinical consensus recommends that the use of CGRP-targeted therapies be restricted to patients with a definitive diagnosis of migraine, particularly those who have failed or are intolerant to conventional preventive treatments (58). In such cases, individualized therapy with CGRP monoclonal antibodies may be considered, provided that a comprehensive cardiovascular risk assessment is conducted prior to initiation. For patients with established CVD or elevated cardiovascular risk profiles, careful pre-treatment evaluation is essential, accompanied by ongoing monitoring of blood pressure, cardiac function, coagulation parameters, and vascular health metrics throughout the treatment course (61, 62). This approach ensures timely adjustment of therapeutic strategies and helps to mitigate potential cardiovascular risks associated with CGRP inhibition.

### 6.4 Opioid analgesics

The use of opioid analgesics in the management of CP comorbid with CVD remains a subject of considerable debate. While opioids such as oxycodone and morphine are effective in alleviating CP, their long-term use has been associated with a range of adverse cardiovascular effects. Emerging evidence suggests that opioids may impair autonomic nervous system function, leading to sympathetic dysfunction, blood pressure variability, and QT interval prolongation, thereby increasing the risk of arrhythmias and cardiovascular events (63). Furthermore, opioids may exacerbate myocardial ischemia through respiratory depression and consequent hypoxemia, further elevating the incidence of adverse cardiovascular outcomes. Given these concerns, the indication for opioid use in CP patients—particularly those at high cardiovascular risk—should be rigorously assessed. Clinicians must carefully balance the analgesic efficacy of opioids against their potential cardiovascular toxicity. Whenever possible, non-pharmacological interventions or alternative, safer analgesic strategies should be prioritized to optimize both pain control and cardiovascular safety.

### 6.5 Antiplatelet agents

Antiplatelet therapy plays a pivotal role in both pain management and cardiovascular prevention strategies. Ticagrelor, a cyclopentyltriazolopyrimidine-class oral antiplatelet agent, reversibly inhibits the P_2_Y_12_ adenosine diphosphate receptor on platelets, thereby preventing platelet aggregation. The PLATO (Platelet Inhibition and Patient Outcomes) trial demonstrated that a 12-month treatment with ticagrelor significantly reduced the composite risk of cardiovascular death, MI, or stroke by 16%, and cardiovascular death alone by 21%, compared to clopidogrel, without a substantial increase in major bleeding risk (64). However, recent scrutiny has been directed at the adjudication of endpoint events in the PLATO trial. A subsequent report questioned the integrity of the event adjudication process, alleging that the committee added 45 MI events to the clopidogrel group while making no corresponding additions in the ticagrelor group (65). This has prompted renewed debate regarding the validity of the trial's conclusions. Despite these controversies, the efficacy of antiplatelet agents in CVD prevention remains well-established. In patients with CP who are at elevated cardiovascular risk, the decision to initiate antiplatelet therapy should be guided by individualized risk-benefit assessments, taking into account both cardiovascular and bleeding risks.

### 6.6 Non-pharmacological therapies

Neuromodulation techniques such as spinal cord stimulation (SCS) and transcutaneous electrical nerve stimulation (TENS) have increasingly been incorporated into the management of CP (66). SCS involves the implantation of electrodes into the epidural space to provide continuous stimulation of the dorsal columns, thereby inhibiting aberrant nociceptive afferent signals and achieving analgesia. In addition to its analgesic effects, SCS has been shown to improve HRV parameters, restore sympathetic-parasympathetic balance, and reduce myocardial oxygen demand, which may in turn decrease the frequency of ischemic episodes. TENS, a non-invasive modality, delivers low-frequency electrical currents through cutaneous electrodes to modulate peripheral nerve activity and inhibit nociceptive transmission. TENS not only suppresses sympathetic-mediated hypertensive responses but also activates the vagus nerve, thereby positively influencing heart rate regulation. These techniques are effective in alleviating refractory pain and may also contribute to cardiovascular protection by modulating autonomic nervous system function, offering novel therapeutic options for CP patients (67).

In recent years, non-pharmacological strategies for pain management have garnered increasing attention for their potential to alleviate CP while concurrently mitigating cardiovascular risks. Psychological interventions such as cognitive behavioral therapy and mindfulness-based stress reduction have demonstrated efficacy in reducing pain perception, as well as anxiety and depression levels. These benefits are mediated in part through attenuation of sympathetic overactivity and suppression of chronic systemic inflammation, thereby improving overall physiological resilience. Evidence suggests that psychological and behavioral therapies may serve as effective non-pharmacologic methods for reducing systemic inflammation, with cognitive behavioral therapy in particular showing promise in enhancing immune function (68). Exercise-based interventions, including tai chi, yoga, and aerobic exercise, have also shown beneficial effects in reducing blood pressure, improving vascular function, and lowering the incidence of CVD. Studies have reported that aerobic exercise can lower systolic blood pressure by an average of 3.84 mmHg and diastolic blood pressure by 2.58 mmHg (69). Moreover, Tai Chi—a traditional form of physical activity—has been demonstrated to exert a positive influence on blood pressure regulation. For CP patients who are intolerant to conventional pharmacological therapies, these exercise and psychological interventions may represent safer and more effective alternatives. Collectively, these non-pharmacological approaches not only aid in pain relief but may also reduce the risk of cardiovascular events by enhancing psychological well-being and physiological function.

**Figure 2:**
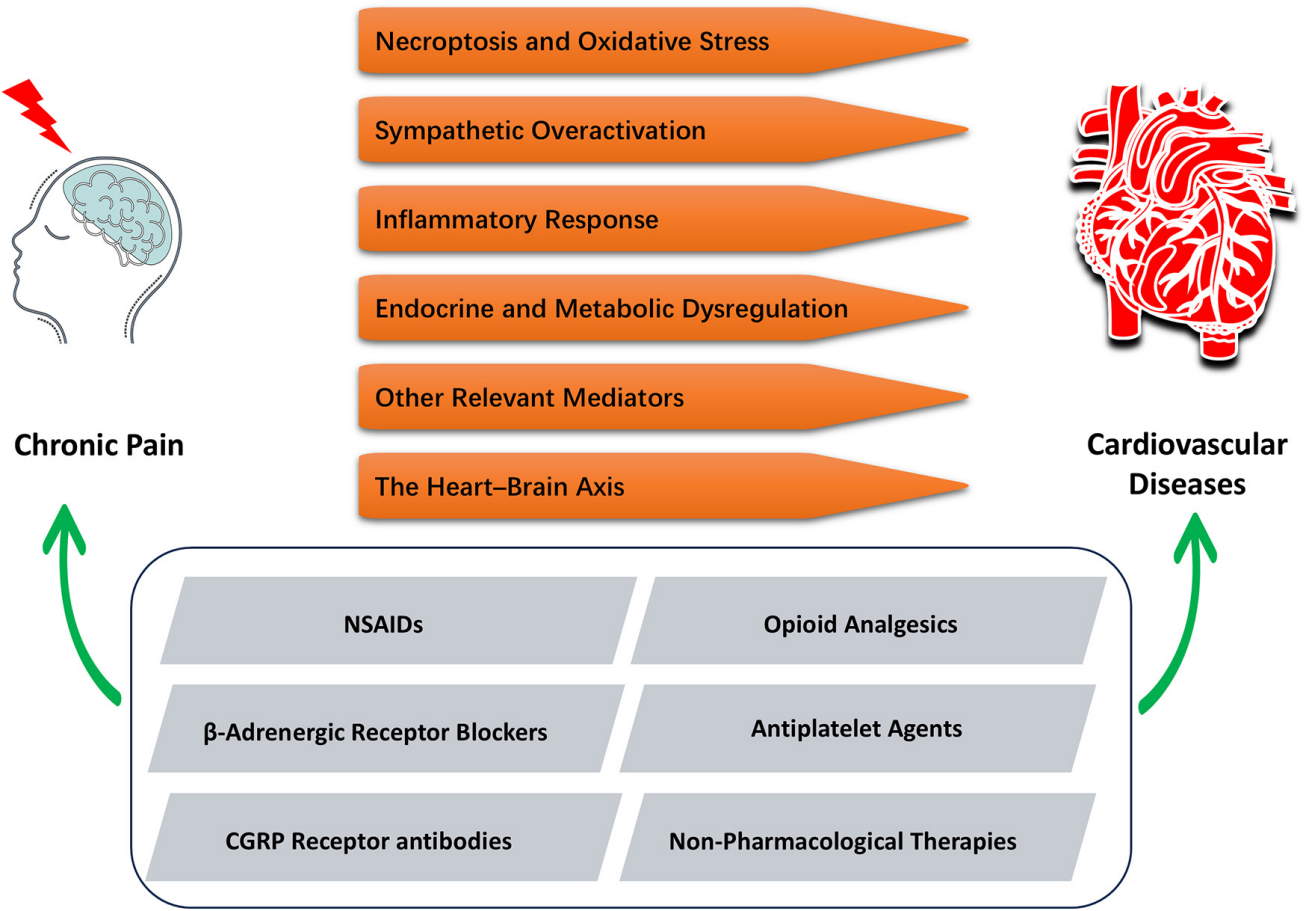
Integrated mechanisms and interventions linking CP and CVD. CP can induce or exacerbate CVD through six distinct mechanisms (in orange). Accordingly, five pharmacological or non-pharmacological interventions (in gray) have been identified that can simultaneously alleviate chronic pain and improve cardiovascular outcomes, offering dual therapeutic benefits.

These findings underscore the importance of an integrated pain management strategy that not only improves quality of life but may also confer cardiovascular benefits. In clinical practice, therapeutic decisions should be guided by individualized risk assessments, balancing the potential benefits and adverse effects of pharmacological agents to optimize outcomes and improve long-term prognosis. Figure 2 illustrates the relevant mechanisms linking CP and CVD, as well as their potential connections in therapeutic approaches.

## 7 Conclusion and future perspectives

In summary, a complex and bidirectional interplay exists between CP and CVD. CP not only significantly impairs quality of life but may also act as an independent risk factor for CVD through mechanisms such as sustained SNS activation, systemic inflammation, neuroendocrine-metabolic dysregulation, and heart-brain axis interactions. Despite these associations, current CVD prevention and management guidelines have largely underrecognized the clinical relevance of CP, potentially underestimating its contribution to cardiovascular risk. From a therapeutic standpoint, the management of CP must strike a balance between effective analgesia and cardiovascular safety. Future research should emphasize personalized treatment strategies tailored to individual risk profiles. A multidisciplinary approach—integrating pharmacologic therapies, physical modalities, and psychological interventions—may offer improved outcomes for patients with CP, particularly those at elevated cardiovascular risk. In conclusion, CP should be recognized as an important modifiable risk factor for CVD. Further mechanistic studies are warranted to elucidate its pathophysiological underpinnings and to inform targeted, evidence-based prevention and treatment strategies. It's important to point out that the way chronic pain affects the cardiovascular system might vary depending on the type of pain, and this deserves more attention in future research. Ultimately, the development of integrated interventions addressing both pain and cardiovascular health may achieve the dual therapeutic goal of alleviating CP while reducing CVD burden.
